# Quantitative Mass Spectrometric Analysis of Ropivacaine and Bupivacaine in Authentic, Pharmaceutical and Spiked Human Plasma without Chromatographic Separation

**DOI:** 10.4137/aci.s2564

**Published:** 2008-05-28

**Authors:** Nahla N. Salama, Shudong Wang

**Affiliations:** 1 National Organization for Drug Control and Research (NODCAR), 6-Abu Hazem Street, Pyramids Ave, PO Box 29, 35521 Giza, Egypt. Email: salama_nahla2004@hotmail.com; 2 School of Pharmacy and Centre for Biomolecular sciences, The University of Nottingham, University Park, NG7 2RD, U.K

**Keywords:** ropivacaine, bupivacaine, 2,6-dimethylaniline, time of flight mass spectrometry, pharmaceutical formulations, spiked plasma

## Abstract

The present study employs time of flight mass and bupivacaine in authentic, pharmaceutical and spiked human plasma as well as in the presence of their impurities 2,6-dimethylaniline and alkaline degradation product. The method is based on time of flight electron spray ionization mass spectrometry technique without preliminary chromatographic separation and makes use of bupivacaine as internal standard for ropivacaine, which is used as internal standard for bupivacaine. A linear relationship between drug concentrations and the peak intensity ratio of ions of the analyzed substances is established. The method is linear from 23.8 to 2380.0 ng mL^−1^ for both drugs. The correlation coefficient was ≥0.996 in authentic and spiked human plasma. The average percentage recoveries in the ranges of 95.39%–102.75% was obtained. The method is accurate (% RE < 5%) and reproducible with intra- and inter-assay precision (RSD% < 8.0%). The quantification limit is 23.8 ng mL^−1^ for both drugs. The method is not only highly sensitive and selective, but also simple and effective for determination or identification of both drugs in authentic and biological fluids. The method can be applied in purity testing, quality control and stability monitoring for the studied drugs.

## Introduction

Mass spectrometry (MS) is one of the most powerful analytical techniques, particularly for pharmaceutical analysis, where good selectivity and high sensitivity are often needed. In the pharmaceutical industry measurements of drugs and their metabolites in plasma are essential for drug discovery and development. The more accurate and rapid these measurements, the more quickly a drug can progress towards regulatory approval. Time-of-flight mass spectrometer (TOF-MS) delivers high sensitivity, resolution, and exact mass measurements. A variety of ion source and software options makes MS a versatile choice for a range of analytical challenges.^1^–^5^

**Figure 1.**
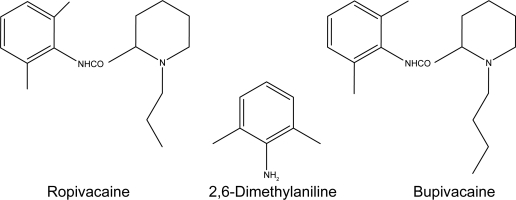
Chemical structures of ropivacaine, bupivacaine and their impurity 2,6-dimethylaniline.

Bupivacaine (Bup), a member of the pipecoloxylidide group (Fig. 1), is the most commonly used local anesthetic. Commercial Bup is the optically inactive racemic (RS) mixture of *R*- and *S*-Bup. Several recent studies have demonstrated that systemic exposure to excessive quantities of Bup result in cardio toxicity due to its high affinity for, and dwell time at, voltagegated sodium channels. A promising alternative to Bup is ropivacaine. Ropivacaine (Rop) is a structural derivative of Bup that differs only by the replacement of the butyl group on the piperidine nitrogen atom of Bup with a propyl group (Fig. 1). The minor structural modification leads to a reduced hydrophobicity and the decreased ability to diffuse into the heart and brain. As a result, Rop has lower systemic toxicity than Bup. In addition, Rop is manufactured as a pure *S*-enantiomer, further lowering the cardiotoxic potential. Both drugs act by blocking the conduction of impulses in target nerve structures, primarily located within the subarachinoid space.^6^–^8^ Several methods using GC with^9^,^10^ or without MS,^11^ high performance liquid chromatography (HPLC) with UV,^12^–^14^ mass spectrometery (MS)^15^–^17^ or amperometric detection,^18^ and capillary electrophoresis^19^ have been developed to analyze the drugs and their impurities in pharmaceutical formulations and/or biological fluids.

The aim of this study is to develop rapid, accurate and simple method for determination of both drugs in authentic, pharmaceutical, spiked human plasma as well as in presence of their impurities without chromatographic separation. The described method was not investigated previously.

In this work we describe how a simple TOF ES-MS analytical method can be used to determine Bup and Rop in authentic and pharmaceutical formulations as well as in spiked human plasma. The technique has many advantages; no need for method development, a short analytical time (1.5 min), and a minimal amount of solvent being required, coupled with high sensitivity, selectivity and exact mass measurements.

## Experimental

### Materials and reagents

Ropivacaine was kindly supplied by AstraZeneca Co., UK, certified to contain 99.00%, CAS No. 132112-35-7. Naropin^TM^ vial containing 7.5 mg mL^−1^ ropivacaine hydrochloride (AstraZeneca Co., UK) was purchased from local market. Bupivacaine was kindly supplied by Al-Debeiky Pharma Co., Egypt, its purity was found to be 99.60% according to BP 2008. Bucain vial containing 5.0 mg mL^−1^ bupivacaine hydrochloride (DeltaSelect, GmbH, Germany) was purchased from the market. Human plasma was kindly donated from volunteers. The following reagents and solvents were purchased and used without further purification: 2,6-dimethylaniline (99.00% Aldich UK, CAS No. 87-62-7), methanol, chloroform (HPLC grade, Fisher Scientific, UK), acetonitrile (LC-MS grade, Reidel-dehaen, UK), ultra pure water (ELGA, UK), formic acid (Sigma-Aldrich, UK), sodium hydroxide (BDH, UK) and hydrochloric acid (Certified Fisher Scientific, UK).

### TLC-separation

Pre-coated TLC plates (10 × 10 cm, aluminium plates coated with 0.25 mm silica gel F254) were purchased from Merck UK. UV-Radiation (Cole-Parmer Instrument, France) detective wavelength was 254 nm.

### TOF-ES MS measurements

The TOF-ES-MS measurements were performed using WATERS––2795 (Waters, UK) equipped with an autosampler injector (10 μL) and Mass Lynx v 4.1. The system was operating in the following regime: electrospray voltage, 3 kv; capillary temperature, 150 °C; sample solution flow rate, 0.1 mL/min. All analysis was performed in the positive ion detection mode. All samples were dissolved in a 50% solution of acetonitrile in water containing 0.1% formic acid.

### Preparation of alkaline degradation products

The degradation products were laboratory prepared by heating 10 mg of bupivacaine hydrochloride in 1 M NaOH (10 mL) for 4 hours on hot plate at temperature 100 °C. The solution was neutralized with 1M HCl then the solution was evaporated and diluted to 10 mL with methanol. Complete degradation was monitored by TLC. The TLC separation was carried out by using chloroform-methanol (9:1 v/v) as the mobile phase.^20^ The R_f_ value is 0.71% ± 0.002% for both drugs. The major alkaline degradation product, namely 2,6-dimethylaniline, which has an R_f_ value of 0.81% ±0.001% as identified by comparison with the reference standard.

### Standard solutions and calibration curves

Stock solutions of ropivacaine and bupivacaine were prepared in methanol at a concentration of 1 mg mL ^−1^, and stored at 4 °C. These were further diluted with 50% aqueous acetonitrile containing 0.1% formic acid to give the appropriate working solutions. Working solutions of each drug were prepared to yield final concentrations of 23.8, 59.5, 119.0, 238.0, 595.0, 1190.0 and 2380.0 ng mL ^−1^ by further dilution with the same solvent. Bupivacaine (2380.0 ng mL^−1^) was used as IS for ropivacaine. Ropivacaine (2380.0 ng mL^−1^), was used as IS for bupivacaine.

### Preparation of spiked human plasma

Aliquots equivalent to 71.4 – 7140.0 ng mL^−1^ of each drug and 7140.0 ng mL^−1^ of the internal standard in 1 mL plasma were sonicated for 5 minutes. Acetonitrile (2 mL) was added and then centrifuged at 7000 rpm for 30 minutes. One milliliter of the supernatant was evaporated.

### Laboratory prepared solutions

Mixtures of ropivacaine and bupivacaine were prepared by mixing different concentrations of each drug with its impurity (2,6-dimethylaniline) and alkaline degradation product, where the ratio 0.1%–10.0% of the mixtures were obtained.

### Procedure

Ten μL each of the above solutions was injected in the TOF-ES-MS under conditions mentioned above. The characteristic m/z ions used for identification and determination of Rop, Bup and 2,6-DMA were *m/z* = 275, *m/z* = 289 and *m/z* = 122 [M +H]^+^, respectively.

### Analysis of pharmaceutical preparations

Milliliters equivalent to 50 mg from the corresponding drug vial were transferred quantitatively into 50 mL volumetric flasks and made up to the volume with methanol. The procedure was completed as mentioned above.

### Calculations

The calibration curves were calculated by unweighted least-squares linear regression analysis of the concentrations of the analyte versus the peak intensity ratio of ions of analyzed substance of ropivacaine (m/z 275) to that of the IS (m/z 289). As for bupivacaine (m/z 289) to that of IS (m/z 275) was used. Concentrations of unknown samples were determined by applying the linear regression equation of the standard curve to the unknown sample’s peak intensity ratio.

## Method Validation

### Limit of quantification

The limit of quantification of the two drugs was defined as the lowest concentration of the calibration curve.

### Precision and accuracy

Precision and accuracy were assessed by assaying freshly prepared solutions of the two drugs in triplicate at three concentration levels; 59.5, 1190.0 and 2380.0 ng mL^−1^. Precision is reported as relative standard deviation (RSD%) of the estimated concentrations and accuracy (% Relative error) expressed as [measured-nominal/nominal × 100].

### Selectivity and specificity

Specificity is the ability of the method to measure the analyte response in the presence of impurity and or degradation products. For specificity determination, synthetic mixtures of different percentages of 2,6-dimethylaniline and degradation products of each drug were added to each pure drug sample. The recovery percent was calculated.

## Results and Discussion

The work includes (1) mass spectrometric identification and determinations of ropivacaine and bupivacaine; (2) generation of the standard calibration curves; (3) identification and determination of drug substances in spiked human plasma; (4) determination of both drugs in presence of alkaline degradation and/or impurity(2,6-DMA); and (5) quantitative analysis of the individual ropivacaine, bupivacaine in their pharmaceutical preparations.

**Figure 2.**
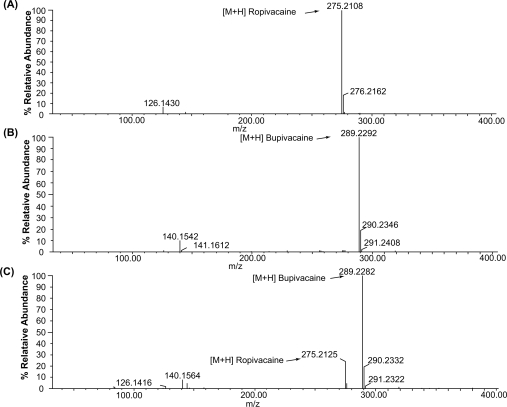
The typical Mass spectra of (**A**) ropivacaine, 2380.0 ng mL^−1^ (**B**) bupivacaine, 2380.0 ng mL^−1^ (**c**) ropivacaine (1190.0 ng mL^−1^, analyte) and bupivacaine (2380.0 ng mL^−1^, internal standard) in 50% aqueous solution of acetonitrile containing 0.1% formic acid.

The mass spectra of ropivacaine and bupivacaine and their internal standards are shown in Figure 2. Under the conditions of TOF ES-MS in positive mode, the spectra displays intense peaks of [M + H]^+^ with ions of the highest mass to charge, e.g. *m*/*z* = 275.2154 for ropivacaine and 289.2226 for bupivacaine, respectively. Linearity range was found to hold good over a determination, concentration range of 23.8 – 2380.0 ng mL^−1^ for both drugs. The results of regression data are presented in Table 1.

**Figure 3.**
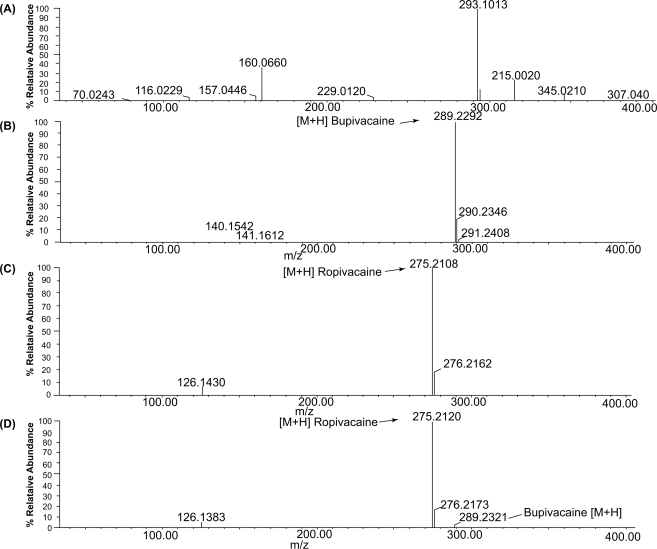
The mass spectra of spiked human plasma (**A**) human plasma, control (**B**) bupivacaine, 1190.0 ng mL^−1^ (**c**) ropivacaine, 1190.0 ng mL^−1^, (**D**) mixture of bupivacaine, 23.8 ng mL^−1^ (analyte) and ropivacaine, 2380.0 ng mL^−1^ (IS) in 50% aqueous solution of acetonitrile containing 0.1% formic acid.

Analysis of studied drugs in spiked human plasma are shown in Figure 3. The linearity was in the range of 59.5–2380.0 ng mL^−1^ for Rop while it is within 23.8–2380.0 ng mL^−1^ for Bup in 1 mL plasma sample, which is the anticipated concentration range in clinical investigation of drug pharmacokinetics. The maximum plasma level of Rop and Bup after different rout of administrations were found to be more than 100 ng mL^−1–21–23^ which could be assessed by the proposed method. The high sensitivity of the proposed method allowed the determination of both drugs in spiked human plasma. Linear regression analysis of the data gives the equations, *A* = 0.02707 C + 0.0082, r = 0.996, for Rop and *A* = 0.5441 C–0.0137, r = 0.999, for Bup. Where *A* is the peak intensity ratio for *m/z* = 275/289 for Rop and 289/275 for Bup, C is the concentration in ng mL^−1^ and r is correlation coefficient.

The data stated in Table 1, indicate that the method is efficient for determination of the studied drugs in biological fluids as there is no significant differences between the results for determinations of both drugs in authentic and spiked plasma samples.

## Validation Data

### Linearity and limit of quantification

Calibration curves for ropivacaine and bupivacaine exhibited good linearity over the concentration range studied (23.8–2380.0 ng mL^−1^) for both drugs in authentic and spiked human plasma as stated in Table 1. From the results, it is clear that there is no interference from the plasma matrix demonstrating the efficiency for determination of the drugs in biological fluids by TOF ES-MS. The limit of quantification (LOQ) was chosen as the lowest calibration standard concentration (23.8 ng mL^−1^) for the studied drugs in authentic and spiked human plasma.

### Precision and accuracy

Table 2 summarizes mean values, precision, and accuracy of intra- and inter-assay analysis. Precision and accuracy were within the ranges acceptable for analytical and bio-analytical purposes. Intra-day precision ranged from 0.60 to 3.61% for ropivacaine while 2.16 to 3.33% for bupivacaine in drug substances. While in spiked human plasma ranged from 1.07 to 7.98% for ropivacaine and from 0.95 to 5.13 for bupivacaine (Table 3). Inter-day precision did not exceed 8.0% over the three level concentrations for three days in drug substances and spiked human plasma. The accuracy of the technique was considered satisfactory, since between-day variation over the concentration range studied was found to be less than 5%.

### Selectivity and specificity

Ropivacaine and bupivacaine are amides expected to alkaline degradation through cleavage of amide linkage with production of 2,6-dimethylaniline. Solutions of alkaline degradations of each were tested by TLC against pure sample of 2,6-dimethylaniline. The same spots of each have the same R_f_ (0.81) of the pure compound. For further confirmation, TOF-ES-MS was carried for each of both compounds. The product obtained from alkaline degradation has *m*/*z* = 122.1935 corresponds to protonated 2,6-dimethylaniline.

**Figure 4.**
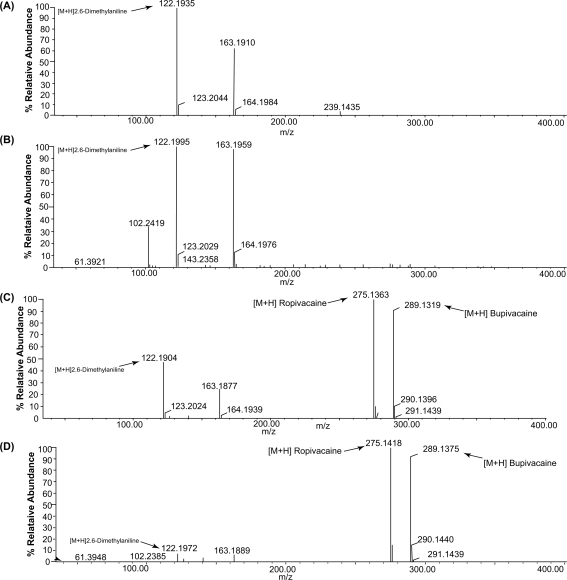
The mass spectra of (**A**) 2,6-dimethylaniline (**B**) Alkaline degradation products (**c**) mixture of ropivacaine, 2380.0 ng mL^−1^ (internal standard), bupivacaine 1190.0 ng mL^−1^ (analyte) and their impurity 2,6-dimethylaniline (**D**) mixture of both drugs with alkaline degradation products, in 50% aqueous solution of acetonitrile containing 0.1% formic acid.

The mass spectrometric determinations of Rop and Bup in the presence of their alkaline degradation products are shown in Figure 4. Synthetic compound 2,6-dimethylaniline was used as control, and in the spectrum (**A**) the ion of the mass to charge (*m*/*z*) 122.1935 corresponding to 2,6-dimethylaniline was identified. The highest ion peak was *m*/*z* = 163.1910 (122 + 41) which might be resulted in acetonitrile solvent interference in the system. The same peaks appeared in the spectrum (**B**) and 2,6-dimethylaniline was the major degradation product of bupivacaine. In addition, a relative low of molecular ion peak at *m*/*z* = 102.2419 was observed which may be assigned as a m-xylene ion. The spectra (**C)** and (**D**) display the intensive ion peaks with *m*/*z* = 122.1935 [M +H]^+^ for 2,6-dimethylaniline clearly indicating 2,6-dimethylaniline to be the major alkaline degradation product of Rop and Bup. The specificity was also assessed by analyzing synthetic mixtures of each drug with its alkaline degradation product in concentration ranging from 0.1 to 10.0%. The results reveal the high selectivity and sensitivity of the method which can determine the impurity in concentration down to 0.1% present in both drugs (Table 4).

### Analysis of pharmaceutical preparations

The method was applied to determine ropivacaine and bupivacaine in Naropin and Bucaine vial respectively. The % RSD was less than 3.0%, indicating the precision of the method, the results are presented in Table 5.

## Conclusion

In this manuscript, we described a newly developed TOF-MS based method for quantitative determination of ropivacaine and bupivacaine in authentic, pharmaceutical dosage forms and the spiked human plasma without chromatographic separation. The strategy of this approach consists in direct multi-ion detection of analytes with reference to internal standards with close structures to the analyte. The method can also be used to identify the degradation products in minute amounts in presence of the corresponding ropivacaine or bupivacaine. The method could be routinely used for quantitative drug analysis in pharmaceutical formulations and biological media as well as for assessing drug purity and stability.

## Figures and Tables

**Table 1.** Linearity, recovery and LOQ of TOF ES-MS assay for ropivacaine and bupivacaine in authentic and spiked human plasma.

**Parameters**	**Ropivacaine**		**Bupivacaine**	
	**Authentic**	**Spiked human plasma**	**Authentic**	**Spiked human plasma**
Linearity ng mL^− 1^	23.8–2380.0	59.5–2380.0	23.8–2380.0	23.8–2380.0
Regression equation				
Slope (b)^a^	0.3645	0.02707	0.6227	0.5441
SE of slope	0.007813	0.011644	0.016894	0.013801
Intercept (a)^a^	0.0133	0.0082	0.0266	−0.0137
SE of intercept	0.009564	0.01303	0.018896	0.016894
Correlation coefficient (r)	0.999	0.996	0.998	0.999
SE of estimation	0.014768	0.023327	0.034049	0.026085
Recovery				
Mean^b^±RSD%	98.83 ± 3.03	95.39 ± 3.64	99.61 ± 3.20	99.96 ± 2.88
LOQ ng mL^− 1^	23.8	59.9	23.8	23.8

^a^ Regression equation, A = a + bc, where A is the peak intensity ratio for m/z = 275.0 /289.0 for Rop, and A is the peak intensity ratio for m/z = 289.0/275.0 for Bup, C is the concentration.

^b^ n = 6.

**Table 2.** Intra and inter-day precision and accuracy of TOF ES-MS assay for ropivacaine and bupivacaine in authentic samples.

**Drug substances**	**Conc. ng mL^−1^**	**Precision^a^ RSD%**		**Accuracy^a^ RE%**	
		**Inter**	**Intra**	**Inter**	**Intra**
Ropivacaine	59.5	3.61	3.57	−4.67	4.59
	1190.0	1.72	2.02	−0.58	−2.19
	2380.0	0.60	1.18	−2.34	2.00
Bupivacaine	59.5	3.33	5.16	2.86	3.12
	1190.0	2.16	2.01	1.05	2.30
	2380.0	2.61	0.96	1.50	3.12

^a^ n = 3.

**Table 3.** Intra and inter-day precision and accuracy of TOF ES-MS assay for ropivacaine and bupivacaine in spiked human plasma.

**Drug substances**	**Conc. ng mL^− 1^**	**Precision^a^ RSD%**		**Accuracy^a^ RE%**		
		**Intra**	**Inter**	**Intra**	**Inter**
Ropivacaine	59.5	7.98	6.50	−3.67	−2.00
	1190.0	3.88	5.75	1.90	3.04
	2380.0	1.07	1.40	0.85	2.50
Bupivacaine	59.5	5.13	4.85	3.66	−1.62
	1190.0	1.95	2.74	−1.07	0.22
	2380.0	0.95	2.99	0.33	0.10

^a^ n = 3.

**Table 4.** Specificity of TOF ES-MS assay for ropivacaine and bupivacaine in authentic samples.

**Degradation products and/or 2,6-DMA%**	**Recovery^a^ % ± RSD%**	
	**Ropivacaine**	**Bupivacaine**
0.1	99.79 ± 2.47	100.04 ± 2.77
1.0	101.05 ± 2.16	100.02 ± 1.04
10.0	98.48 ± 1.29	98.18 ± 1.67

^a^ Mean of four different experiments.

**Table 5.** Results for the determination of ropivacaine and bupivacaine in pharmaceutical formulations by the proposed TOF ES-MS method.

**Preparations**	**TOF ES-MS**	
	**Mean recovery^a^ %**	**RSD%**
Naropin vial, 7.5 mg mL^−1^ ropivacaine hydrochloride	102.75	1.75
Bucain vial, 5.0 mg mL^−1^ bupivacaine hydrochloride	100.33	2.51

^a^ Mean of five experiments.
